# Preparation, Modification, and Application of Ethylene-Chlorotrifluoroethylene Copolymer Membranes

**DOI:** 10.3390/membranes14020042

**Published:** 2024-02-01

**Authors:** Zhangbin Liao, Qian Wang, Qiuyueming Zhou, Zhaoliang Cui, Zhaohui Wang, Enrico Drioli

**Affiliations:** 1 State Key Laboratory of Materials-Oriented Chemical Engineering, College of Chemical Engineering, Nanjing Tech University, Nanjing 210009, China; zbliao1997@163.com (Z.L.); chelseawang@njtech.edu.cn (Q.W.); 202061104098@njtech.edu.cn (Q.Z.); 2 National Engineering Research Center for Special Separation Membrane, Nanjing Tech University, Nanjing 210009, China; 3 Jiangsu National Synergetic Innovation Center for Advanced Materials (SICAM), Nanjing Tech University, Nanjing 211816, China; 4 Research Institute on Membrane Technology, ITM-CNR, Via Pietro Bucci 17/C, 87036 Rende, Italy; e.drioli@itm.cnr.it

**Keywords:** ethylene-chlorotrifluoroethylene, membrane, grafting, modification

## Abstract

Ethylene-chlorotrifluoroethylene (ECTFE) was first commercialized by DuPont in 1974. Its unique chemical structure gives it high heat resistance, mechanical strength, and corrosion resistance. But also due to these properties, it is difficult to prepare a membrane from it by the nonsolvent-induced phase separation (NIPS) method. However, it can be prepared as a microfiltration membrane using the thermally induced phase separation (TIPS) method at certain temperatures and with the selection of suitable solvents, and the use of green solvents is receiving increasing attention from researchers. The surface wettability of ECTFE membranes usually needs to be modified before use to strengthen its performance to meet the application requirements, usually by graft modification and surface oxidation techniques. This paper provides an overview of the structure of ECTFE and its preparation and modification methods, as well as recent advances in its application areas and prospects for the future methods of preparing high-performance ECTFE membranes.

## 1. Introduction

**Figure 1:**
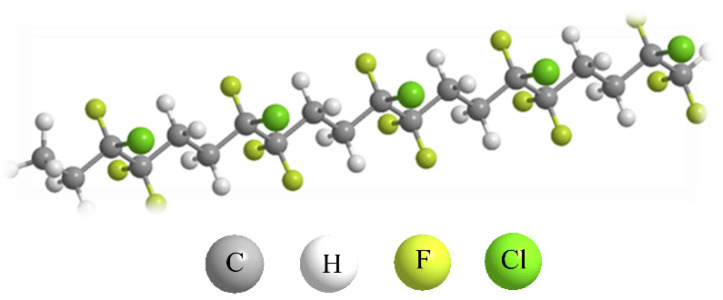
ECTFE molecular structure.

Ethylene-chlorotrifluoroethylene (ECTFE) is a copolymer composed of alternating monomer units of ethylene and chlorotrifluoroethylene. Its chemical structure is shown in Figure 1. The F atom has strong electronegativity and low polarization, and the C-F bond exhibits one of the strongest chemical bond energies within the structure, reaching up to 485 kJ/mol. At the same time, multiple F atoms are connected to the C atom, which further strengthens the C-F bond energy and creates its excellent performance [1]. The regular pattern of these monomer units results in a well-defined structure, which gives ECTFE its consistent and predictable properties.

**Figure 2:**
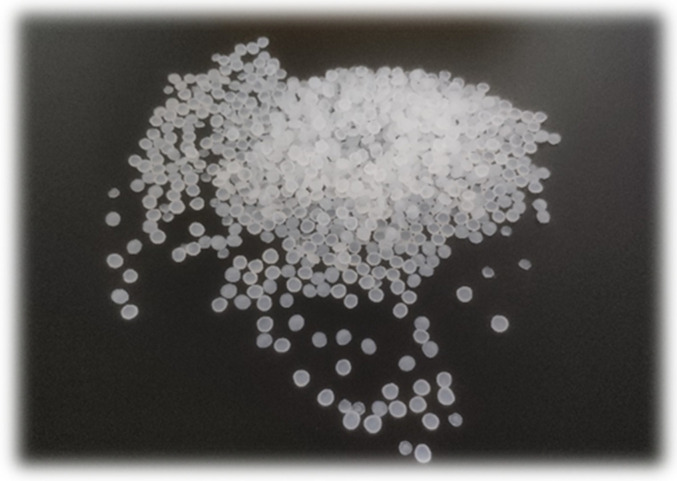
ECTFE particles from the Solvay company.

This unique type of fluorinated elastomer was first commercialized by DuPont in 1974 under the trade name Halar^®^. In 1986, Applied Chemical Organization transferred ECTFE products and production technology to Ausimont USA Inc. (Morristown, DE, USA). In 2001, the Solvay Group of Belgium acquired Ausimont, thereby becoming the sole producer of ECTFE. Figure 2 shows ECTFE particles from the Solvay company (Brussels, Belgium), demonstrating their uniform size and smooth surface.

ECTFE exhibits high chemical corrosion resistance, mechanical strength, and thermal resistance; exhibits low capacitance, flammability, refractive index, and surface energy (neither oil-wet nor water-wet); is especially inactive to various solvents, including hydrocarbons and various acids and bases, with no solvent able to attack it below 120 °C [2,3,4,5]. In terms of resistance to strong alkalis and acids, high-temperature resistance, and chemical resistance, ECTFE is even superior to other fluorinated materials, such as polytetrafluoroethylene (PTFE) [6,7] and poly (vinylidene fluoride) (PVDF) [8,9], and is an ideal material for preparing high-performance porous membranes.

The polymer’s low polarizability and strong electronegativity also contribute to its excellent thermal stability and low coefficient of friction, allowing it to be used in high-temperature and wear-resistant applications. Additionally, due to the presence of chlorine atoms, ECTFE has stronger resistance to water vapor, hydrogen chloride, and chlorine gas than ordinary fluoropolymers. Its chlorine permeability is the best among all fluoropolymers, so it is widely used in some harsh environments exposed to chlorine. Even if ECTFE is exposed to ultraviolet light for a long time, its performance remains unchanged and can be used in the construction industry, such as in anti-UV coatings. It is commonly used as a protective coating, including for pipeline protection and corrosion prevention [10,11]. For example, ECTFE is frequently used as a coating for stainless steel exhaust pipes in a range of industrial applications (including clean rooms) to protect them from the corrosive effects of various airflows. When compared to PTFE coating, the adhesion and hardness of an induced draft fan impeller coated with ECTFE are twice as good when exposed to hydrofluoric acid containing special corrosive substances. Furthermore, ECTFE’s exceptional properties also make it an excellent material for use as an anticorrosion membrane resin on the surface of solar photovoltaic modules. In these applications, ECTFE resin has demonstrated exceptional corrosion resistance, weather resistance, and chemical resistance. This makes it an excellent long-term solution for protecting photovoltaic modules from environmental degradation and corrosion. However, it should be noted that the research on ECTFE as a porous membrane is still in its early stages. Despite its potential for a range of practical applications, further research is required to optimize its performance and explore its full potential.

## 2. Fabrication of ECTFE Membrane

Microfiltration membranes are usually prepared using thermally induced phase separation (TIPS) [12,13,14,15,16] or nonsolvent-induced phase separation (NIPS) [17,18,19,20,21,22,23].

TIPS is a relatively new method of preparing polymer microporous membranes proposed and patented by Castro in 1981. Its process and principle are above the melting point of the polymer; the polymer will be dissolved in a high-boiling-point and low-volatility diluent to form a homogeneous solution. It is then cooled down. During cooling, the system undergoes phase separation. This process is divided into two categories, solid–liquid phase separation (referred to as S-L phase separation) and liquid–liquid phase separation (L-L phase separation). The appropriate process conditions are controlled, and after phase separation, the system forms a two-phase structure with the polymer as the continuous phase and the diluent as the dispersed phase. At this point, the appropriate volatile reagent (i.e., extractant) is selected to extract the diluent in order to obtain a certain structure shape of the polymer porous membrane.

Using NIPS, the polymer is dissolved in a solvent to form a homogeneous solution, and then a reagent that is more soluble with the solvent (known as the extractant) is slowly added to extract the solvent, forming a two-phase structure with the polymer as the continuous phase and the solvent as the dispersed phase; then the solvent is removed to obtain polymers with a certain pore structure.

Compared with NIPS, TIPS has many advantages: (1) TIPS promotes phase separation of polymer solutions through faster heat exchange rather than slow solvent–nonsolvent exchange. (2) TIPS avoids the disadvantage of NIPS, that is, a low porosity due to the existence of the solvent–nonsolvent exchange, which leads to part of solvent participating in polymer gelation during the membrane-forming process. (3) TIPS can be used for the preparation of crystalline polymer microporous membranes that are difficult to prepare with NIPS. (4) TIPS has fewer influencing factors than NIPS and is easier to be controlled. (5) A variety of microstructures can be obtained by TIPS such as open-pore, closed-pore, isotropic, anisotropic, and asymmetric, etc.

To date, no organic solvent is able to dissolve ECTFE at room temperature. Therefore, some common membrane preparation methods, such as NIPS [12,13,14,15,16,17,18], cannot be used to prepare ECTFE membranes. However, with increasing temperature, especially when the temperature is above the melting point of ECTFE, ECTFE can form homogeneous solutions with some diluents, which provides the basis for the preparation of ECTFE membranes by TIPS [4,12,13,14,15,16]. One can control the pore size of ECTFE membranes when using TIPS by adjusting the cooling temperature and selecting the appropriate solvent. The main problem in the preparation of microfiltration membranes by the TIPS method is the selection of diluents.

**Figure 3:**
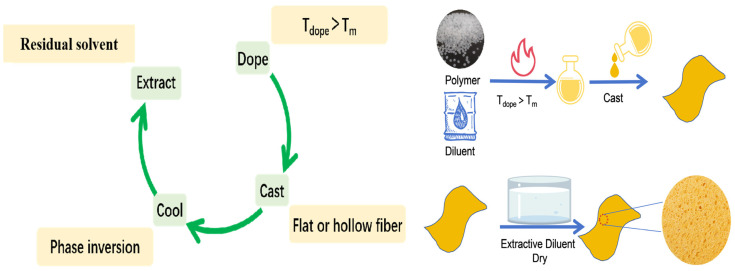
The procedure for preparing ECTFE membranes by TIPS. (T_dope_ is the temperature of casting solution, T_m_ is the temperature of melting temperature).

During TIPS, the polymer is dissolved in a diluent at a temperature above its melting point to form a homogeneous solution [24,25,26,27,28,29,30]. By ensuring the uniform distribution of the polymer in the solution, a high-quality membrane can be made. The homogeneous solution is subsequently cooled to induce the phase separation. The main steps of preparing a microfiltration membrane by TIPS include solution preparation (continuous or intermittent preparation), membrane configuration (plate or hollow-fiber as shown in Figure 3), and post-treatment [31,32]. The specific steps are as follows: (1) Mix the polymer with a high-boiling-point and low-molecular-weight liquid or solid diluent at a high temperature to form a homogeneous solution. (2) Cast the solution into the desired shape (flat, hollow, or tubular). (3) Cool the solution to induce phase separation. (4) Remove the diluent (cosolvent extraction). (5) Remove the extractant (by evaporation) to obtain the porous structure.

Table 1 lists several studies on the production and applications of porous ECTFE membranes. It is crucial to select an appropriate diluent for the ECTFE polymer, as this process can be challenging. The polymers must be dissolved in solvents at a temperature of 250 °C, which requires the use of solvents with high boiling points, flash points, and melting points that are higher than the melting point of the polymer. Moreover, it is paramount that these solvents are safe for human use and the environment. These constraints hinder the advancement and industrial application of ECTFE polymers in the membrane industry.

**Figure 4:**
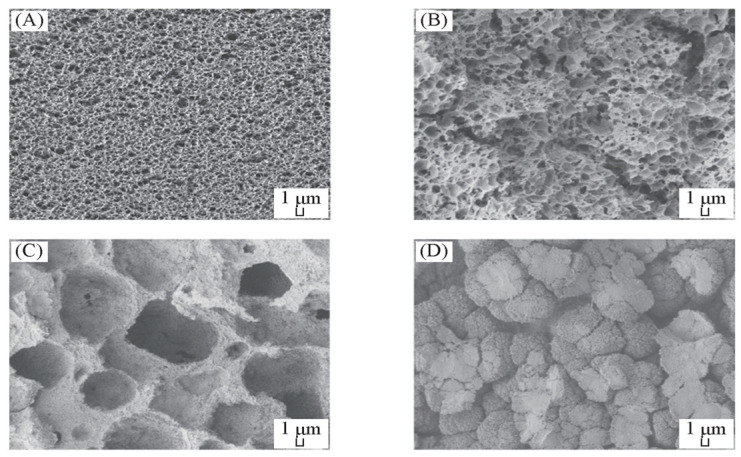
The cross-section structure of ECTFE membranes prepared with different solvents: (**A**) DEP, (**B**) DBP, (**C**) BS, (**D**) DBS. Reprinted from Ref. Zhou 2012 [14]. Copyright permission has been obtained from the Publisher Editorial Office.

The ECTFE membrane was first prepared by the TIPS method in 2002 by Ramaswamm et al. In that study, a surface-dense ECTFE membrane was successfully prepared with DBP as a single dilution. The dense layer was successfully eliminated and the porosity was improved [11]. In 2010, Roh et al. improved the membrane preparation process and conducted a detailed study on the effects of casting fluid composition, quenching temperature, membrane thickness, bore fluid composition, and traction force on the structure and performance of an ECTFE membrane when preparing the membrane using the TIPS process [12]. However, the cross-sectional structure of the ECTFE membrane prepared with DBP as the diluent was incomplete. Fundamentally, this structure was obtained using DBP because the system had a relatively narrow liquid–liquid (L-L) phase separation region, resulting in the short L-L phase separation process, resulting in the formation of a disordered spherulite structure. The effect of the interaction parameters between ECTFE and different solvents on the L-L phase separation region of the ECTFE solvent system was studied by Zhou et al. [14]. The Flory–Huggins interaction parameter reflects the change in the interaction energy of the polymer molecules during mixing with the solvent and is denoted by χ. From the polymer solution thermodynamic theory for derivation, it can be seen that the value of the polymer–solvent interaction parameter χ can be used as a semiquantitative judgment of the solvent superiority. If χ is greater than 0.5, the polymer is generally insoluble; if χ is less than 0.5, the polymer can be dissolved, that is, the smaller the value, the better the solvent’s ability to dissolve. Therefore, the value can be used as a basis for determining whether a polymer and a solvent system are miscible. The selected solvents and interaction parameters with the ECTFE are shown in Table 2. The cross-section structure of the ECTFE membrane prepared with the corresponding solvent is shown in Figure 4. It can be observed that the order of the interaction parameters between ECTFE and each solvent is χ_ECTFE/DMP_ > χ_ECTFE/DEP_ > χ_ECTFE/DBP_ > χ_ECTFE/BS_ > χ_ECTFE/DBS_. Increasing interaction parameters change the cross-sectional structure of the membrane from a spherulite structure to a cellular structure or a continuous structure. The larger the interaction parameter χ, the less the compatibility of the polymer with the solvent, indicating the wider the L-L phase separation area. At appropriate cooling rates, the ECTFE cross section will present a double continuous structure.

However, in the published papers, the cross sections of ECTFE membranes prepared using a single diluent in the TIPS process present either closed cellular structures with good mechanical properties but almost no pores or spherulite structures with poor mechanical properties, which is not sufficient for practical applications.

**Figure 5:**
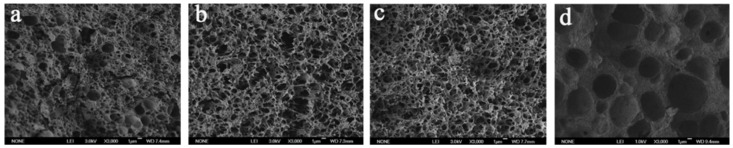
The cross-sectional structure of the ECTFE membranes prepared by adding different nonsolvents: (**a**) HDC; (**b**) TPP; (**c**) OA; (**d**) TEG. Reprinted from Ref. Zhou 2013 [41]. Copyright permission has been obtained from the Publisher Editorial Office.

The effect of mixed solvent in ECTFE membranes prepared by the TIPS method on the microfiltration membrane structure and properties was also thoroughly investigated by Zhou et al. [41,42]. The investigators also studied the effects of different nonsolvents on the crystallization trends of the ECTFE/DBS systems. The solvent parameters of the four nonsolvents and the interaction parameters with ECTFE χ are shown in Table 3, and their membrane morphology is shown in Figure 5. The solubility parameter distances between ECTFE and each nonsolvent are as follows: χ_(ECTFE-TEG)_ > χ_(ECTFE-TPP)_ > χ_(ECTFE-OA)_ > χ_(ECTFE-HDC)_ (TEG is triethylene glycol, TPP is triphenyl phosphate, OA is an oxidized amine, HDC is hexamethylene diamino carbamate). The solubility parameters of DBS are relatively close to those of TPP and OA, indicating that DBS has good compatibility with TPP and OA. In both cases, the ECTFE membrane exhibits a bicontinuous structure. The other two remain microporous cellular structures because when the solvent compatibility is poor, the polymer is cooled first during the binary diluent cooling process, thus forming an irregular cross-sectional structure. Zhou’s work [41] offers us profound inspiration. The use of a single solvent in the preparation of polymer membranes often has numerous limitations, making it challenging to expand the polymer–solvent system. By applying the theory of the solubility parameter, the L-L separation area can be separated according to the theory of the solubility parameter, adjusting the interaction between polymers and solvents. This approach enables us to find suitable solvents and additives.

To prepare high-performance ECTFE membranes, most studies are limited to a temperature of around 250 °C, which is very close to the decomposition temperature of ECTFE. At this temperature, ECTFE will be rapidly oxidized and degrade, and it will also cause diluent volatilization, resulting in harm to the environment and researchers. Before ECTFE membranes were prepared at relatively low temperatures by Simone [15] et al. and Drioli [33] et al., researchers gradually turned their attention to how to prepare ECTFE membranes in a more environmentally friendly manner. In Simone et al.’s work, they found that N-methylpyrrolidone (NMP) can dissolve ECTFE at 180 °C [15].

What is more, the solvents for dissolving ECTFE mostly use aromatic compounds with high boiling points, which are generally toxic and will cause harm to human health and the natural environment. Therefore, the development of environmentally friendly solvents for the TIPS method has attracted more and more attention from researchers [43,44]. Xu [5] et al. and Liu [40] et al., respectively, selected the environmentally friendly diluents ATBC and TOTM as a single solvent to prepare the ECTFE membrane. Liu et al. chose TOTM as the solvent, and a significant L-L phase separation was observed for the bicontinuous structure when the polymer concentration was 15%. As the polymer concentration increased, the ECTFE membrane structure changed from a bicontinuous structure to a stacked-block structure. During the 30 h membrane distillation process, the ECTFE membrane at 15% TOTM achieved a retention of 23.09 kg·m^−2^·h^−1^ and a 99.9% rejection rate. The ECTFE membrane maintained great salt protection at a running-feed solution concentration ratio of 3.49 (i.e., 12.1 Wt.% concentration).

As can be seen from the development of ECTFE membranes prepared by the TIPS method, researchers have begun to pay attention to the preparation of high-performance membranes in a safe and environmentally friendly way. The choice of diluent has also changed from toxic phthalic acid reagents to green solvents, such as ATBC and TOTM, etc. Finding ways to successfully use green solvents to prepare high-performance ECTFE membranes under low-temperature conditions to effectively excavate the potential of this high-performance partial-crystalline fluorine-containing material in the field of the membrane is in line with the development concept of the green chemical industry.

## 3. Modification of ECTFE Membrane

In some practical applications, the surface wettability of microfiltration membranes needs to be modified. The most commonly used ECTFE membrane modification methods are graft modification and surface-coating modification. Graft modification involves the chemical bonding of monomers or polymers to the groups on the membrane surface, resulting in changes in surface energy and wettability [45,46,47,48,49,50,51]. Surface-coating modification [52,53,54,55,56,57,58,59], on the other hand, involves depositing a layer of thin film onto the membrane surface, which can significantly change its surface properties. Therefore, it is necessary to select the appropriate modification method according to the application requirements.

### 3.1. Graft Modification

Grafted modifications are generated by the reaction of suitable branches or functional-group side groups with macromolecular chains. Grafted copolymers are characterized according to their composition, structure, length, and number of backbones and branches. Graft modification can give the polymer two or more sharply opposite properties. Short-chain branched grafts resemble random copolymers, while long-chain branched grafts resemble blends. Through copolymerization, two polymers with different properties can be grafted together to form grafts with special properties. Therefore, polymer graft modification has become a simple and effective method to expand the scope of polymer application and improve the material properties. Abdel-Hady [35] grafted a binary monomer (styrene and vinyl pyrrolidone) onto an ECTFE membrane with different gamma radiation doses. When the ratio of styrene to vinyl pyrrolidone was 1:1, the applicability of a proton exchange membrane fuel cell (PEMFC) was explored by its ion exchange performance, water absorption energy, proton conductivity, free volume size, membrane thickness, and tensile strength. At 75 °C, depending on the highest graft membrane value of the fuel cell performance, it was more durable than the compressed Nr.118 (Commercial Nr.118 membranes with 50 mm thickness, purchased from Optco, Egypt) and lasted for 450 h. In another study, Abdel-Hamed [39] grafted styrene onto a commercial ECTFE membrane and then sulfonated the membrane. Diluting the styrene on ECTFE with a solvent mixture of methanol and dichloromethane (1:1) can effectively promote the grafting reaction. ECTFE-g-PSSA has low cost and high conductivity, which can be a better alternative to Nafion for direct methanol fuel cells.

**Figure 6:**
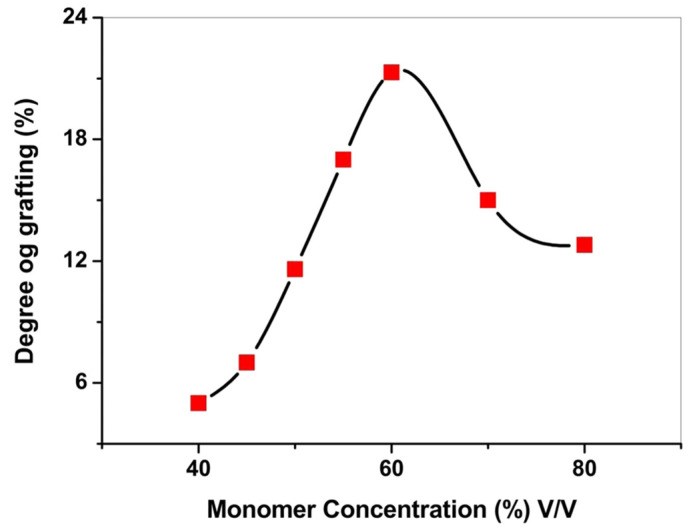
The effect of monomer concentration on the degree of grafting. Reprinted from Ref. Abdel-Hamed 2018 [39].

At the same time, Abdel-Hamed studied the effect on the grafting degree when styrene was diluted using different solvents under controlled parameters, as shown in Table 4. This is due to the cumulative effect of the swelling of the ECTFE membrane and the change in the partition coefficient of styrene between the membrane and the external liquid phase, which leads to an increase in the local styrene concentration around the grafting site. The trend of the influence of the volume fraction of the monomer in the solvent on the grafting rate was also explored, and the results are shown in Figure 6, where the grafting rate first increased with the increase in monomer concentration until it started to decrease after reaching the maximum value. This is due to the swelling behavior of ECTFE during the grafting process and the availability of monomer to the grafting site. When increasing the monomer concentration, the number of monomers and the diffusion of monomers to the grafting sites increased, and the grafting rate increased. Styrene itself caused swelling of the polymer, but the swelling effect was lower than that in the presence of methanol and dichloromethane; therefore, the spreading of monomers to the grafting site through the activation radicals decreased, resulting in a lower level of grafting.

### 3.2. Surface Oxidation

Anari [60] formed a thin hydrophilic layer on the surface of the ECTFE membrane through simple surface oxidation modification. The existence of O-functionalized hydrophilic groups on the membrane was confirmed by energy-dispersive X-ray spectroscopy (EDX) and X-ray photoelectron spectroscopy (XPS). The effect of modification temperature and the surface oxidation time on the properties of the obtained membrane were also systematically investigated. Properly increasing the modified temperature and surface oxidation time can improve the membrane’s antifouling ability. After surface modification through oxidation, the thickness and the water contact angle of the membrane decreased, and the modified membrane showed higher initial permeate flux compared to a virgin membrane. The results show that the induction of optimal hydrophilicity can successfully reduce organic contamination, and membrane initial flux recovery of over 90% of the MD cycle can be achieved after simple membrane cleaning with water.

The above results indicate that surface oxidation technology can optimize hydrophilicity without increasing membrane thickness and the membrane’s ability to resist organic pollution without affecting other performances of the membrane.

## 4. Application of ECTFE Membrane

Currently, the research on membrane separation technology aims to improve the mechanical strength, chemical resistance, and pollution resistance of the membrane. The exploration of emerging membrane processes, such as membrane contactor processes, membrane reactor processes, and membrane distillation, has also attracted wide attention from researchers. The traditional separation process is energy-consuming and time-consuming, and the world’s demand for membrane separation technology accelerates its development and puts forward new requirements to reduce the cost, improve the separation effect, and expand the application field of membranes. ECTFE has excellent corrosion resistance, high-temperature resistance, and chemical resistance, so it has a broad application prospect.

### 4.1. ECTFE Membrane for Membrane Condensers

Membrane condensers are a new membrane separation technology with simple equipment and mild operating conditions [61,62,63,64,65,66,67]. This technology has wide application potential and value in water vapor recovery, chemical concentration, and recovery processes [68].

In 2013, Drioli et al. [69] proposed the working principle of a membrane condenser based on hydrophobic membranes. The principle is that the water-containing gas enters the membrane module. The water vapor in the gas condenses due to the temperature difference between the wet gas and the surface of the membrane module. Therefore, water condenses on the membrane surface without entering the membrane hole, and the dehydrated gas passes through the membrane hole and becomes dehumidified gas. The most prominent feature of membrane condensation is the use of hydrophobic membranes, which not only avoids the resistance of water droplets but also promotes the condensation of water vapor. Membrane condensation can also control and reduce pollutants in circulating water and improve the purity of water.

In 2014, Drioli’s [33] team used the green solvent triglyceride (GTA) as a diluent to make an ECTFE membrane. The membrane prepared by the TIPS method showed an asymmetric spongelike microporous structure. The thickness of the ECTFE flat-sheet membrane was about 110 μm. The average pore size was approximately 46 nm, and porosity was 83%. The feed temperature and feed flow were changed to selectively recover water from the gaseous state, and the process simulation was studied. The results showed that both polymers had similar recovery rates of 35% and 55%, respectively, well above the 20% required for the plant to be self-sufficient.

The above results indicate that although the productivity of flat membranes is lower than that of tubular or capillary membranes due to the reduced effective surface area, similar results were achieved in terms of water recovery. Therefore, ECTFE membranes are considered promising candidates for membrane condensers.

### 4.2. ECTFE Membrane for Membrane Distillation

Membrane distillation (MD) has recently gained prominence as a membrane separation process utilizing nonisothermal physical separation technology. In this technology, a hydrophobic microfiltration membrane is employed to segregate the aqueous solution [70,71,72,73,74,75]. By introducing a temperature difference across the membrane, a vapor pressure disparity is created, propelling vapor through the hydrophobic porous membrane. A cold trap is utilized to accumulate the steam on the osmosis side. This technique has been widely used in the desalination field.

The MD process can be categorized into four fundamental types based on variations in the steam-driving mechanism through the hydrophobic porous membrane and the steam-collection approach on the osmosis side. These four types, namely, direct contact membrane distillation (DCMD), air gap membrane distillation (AGMD), sweeping gas membrane distillation (SGMD), and vacuum membrane distillation (VMD), significantly impact the separation efficiency and operational costs of porous membranes in MD applications.

Xu et al. [5] employed the environmentally friendly ATBC as a sole diluent to fabricate the ECTFE membrane, which exhibited superior hydrophobicity and a higher fluid entry pressure (LEPw). Table 5 illustrates the average pore size and porosity of ECTFE porous membranes synthesized at different polymer concentrations. In the context of VMD, which is characterized by lower heat-transfer losses and reduced boundary-layer mass-transfer resistance compared to other MD types, the ECTFE membrane displayed commendable permeate-flux and salt-rejection rates.

**Figure 7:**
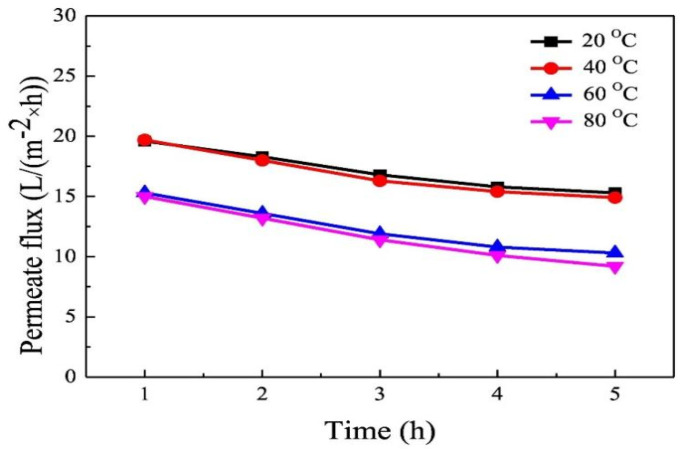
Permeate flux as a function of the running time of the prepared membranes with different quenching temperatures (polymer concentration was 30 Wt. %). Reprinted from Ref. Xu 2019 [5].

The membrane’s pore size and porosity were found to decrease with an increase in quenching temperature, resulting in a gradual reduction in the stable permeation flux. Table 6 provides insights into the average pore size and porosity of ECTFE membranes fabricated at different quenching temperatures. Remarkably, at a quenching temperature of 80 °C, the permeate flux reached 22.3 L/(m^2^·h), with an impressive salt-rejection rate of 99.9%. These outcomes underscore the promising application potential of ATBC-prepared ECTFE membranes in VMD, as illustrated in Figure 7.

As the concentration of ECTFE increased, the average pore size and porosity of the prepared porous membrane decreased, which is due to the increase in spherulite density and the reduction in voids between spherulites.

As the quenching temperature increased, the average pore size decreased and the porosity slowly increased. This is because increasing the quenching temperature intensified the migration of the diluent. Although the pore size increased, the polymer concentration in the middle of the membrane relatively increased, forming a denser spherical structure, resulting in a decrease in the total pore size. At the same time, the evaporation of the diluent intensified, causing a slow increase in the porosity of the membrane.

It could be seen from the Figure 7 and Table 7 that increasing the quenching temperature can reduce the permeability of the membrane and also demonstrate the great potential of ECTFE flat membranes in MD applications.

### 4.3. ECTFE Membrane for Organic-Solvent Filtration

**Figure 8:**
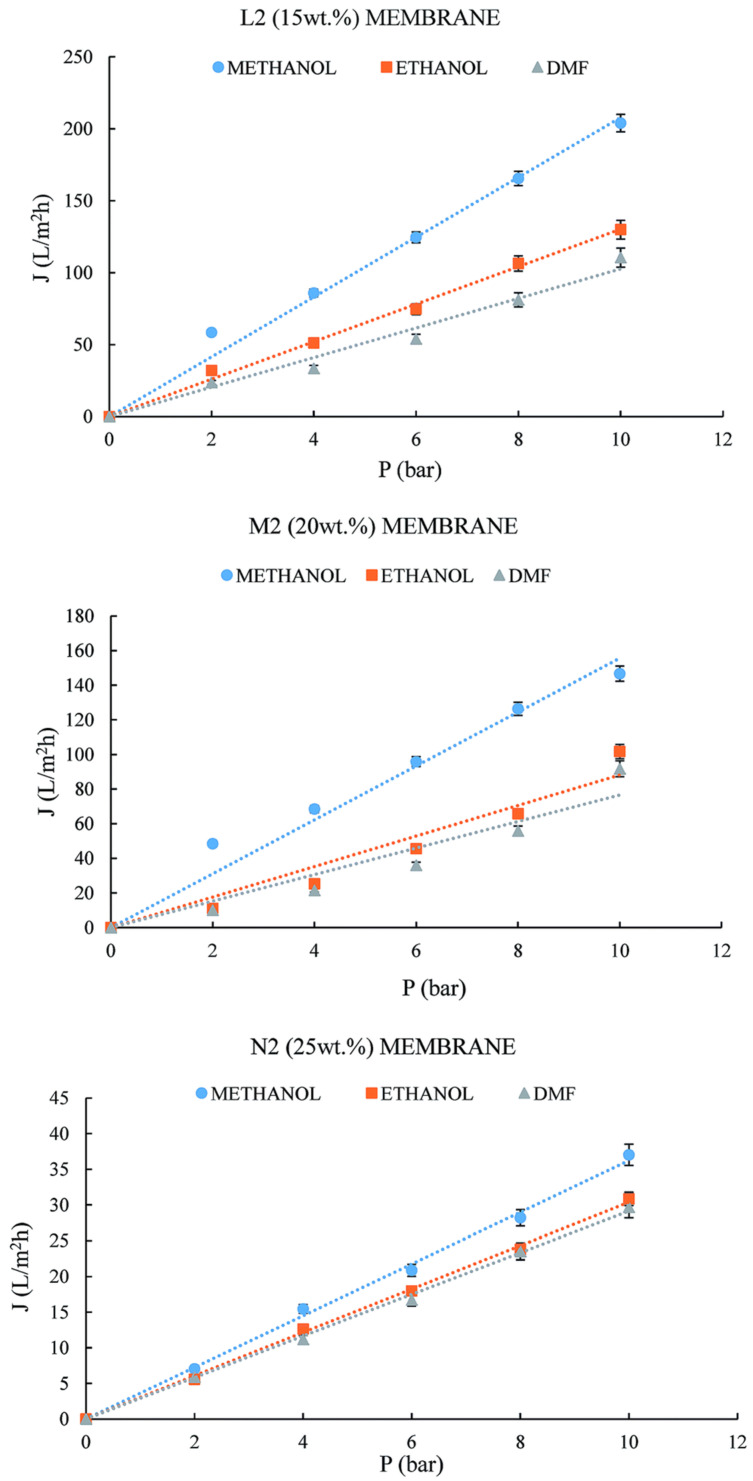
Solvent permeability trends with three different organic solvents and ECTFE membranes. Reprinted from Ref. Ursino 2016 [37].

Ursino [37] prepared a low-melting-point Halar^®^ ECTFE (LMP ECTFE) and utilized a green and nontoxic diluent (DEA) (DEA is a diethylhexyl ester) to prepare dense and porous asymmetric flat membranes. The average pore size and porosity of porous membranes are shown in Table 8. The results of the solubilization test after 192 h showed that the ECTFE membranes had good solvent resistance; the porous membranes could be used for pure solvent filtration experiments such as methanol, ethanol, and dimethylformamide; and the experimental fluxes increased with the increase in pressure. This study shows the potential of the ECTFE membrane to separate organic solutions, as shown in Figure 8. The permeability of the membranes L2, M2, and N2 was prepared using 15-20-25 Wt.% of polymer.

As shown in Figure 8, the permeate flux increased linearly when increasing the pressure. This result is reasonable considering the molecular weight and viscosity of both solvents.

Simone [15] prepared ECTFE membranes by the TIPS method using NMP as a solvent. The effect of four additives, glycerol triacetate (GTA), triethyl citrate (TEC), diethyl succinate (DBI), and diethyl adipate (DEA), on the ECTFE membrane structure and performance was investigated. These asymmetric dense membranes consisted of a skin layer facing the air side and a support layer composed of spherulitic structures. Solvent uptake tests were carried out on the ECTFE membranes using different solvents. Organic-solvent/water mixtures and organic-solvent/organic-solvent mixtures were used as test systems. Additionally, a permeation evaporation test was also performed on the toluene/water system. The thickness of the dense layer of the ECTFE films prepared with different diluents was not the same. The obtained results suggest that the prepared ECTFE dense membranes are very promising candidates for organic-solvent pervaporation and also for nanofiltration.

The above research indicates that when filtering organic solvents, on the one hand, they are affected by the physical properties of the solvent itself, such as molecular weight, density, and surface tension, etc. On the other hand, as the polymer concentration increases during the membrane-making process, its allowed permeability also decreases, and it also has good screening ability for organic solvents, indicating its potential application in the field of organic-solvent filtration.

### 4.4. ECTFE Membrane for Proton Exchange Membrane Fuel Cell (PEMFC)

Over the past decades, PEMFC has had the advantage of high power density, simple operation, high energy conversion efficiency, and near-zero harmful emissions. APEMFC consists of an anode, a cathode, and a proton exchange membrane. The anode is where the hydrogen fuel is oxidized, and the cathode is where the oxidant is reduced. Both electrodes contain a catalyst that accelerates the electrochemical reaction at the electrodes. H^+^ is allowed to pass through while the electrons lost by H_2_ pass through the wire. When working, it is equivalent to a direct current power supply. The anode is the negative terminal of the power source and the cathode is the positive terminal of the power source. According to the principle of fuel cells, the membrane must have a good proton conductivity, high dielectric constant, low methanol and gas permeability, high chemical equilibrium, and mechanical properties [35]. Abdel-Hamed [39] used a solution-grafting technique to graft styrene onto commercial ECTFE membranes and then sulfonated the membranes. He studied the effect of various diluting solvents and monomer concentrations on the degree of grafting (DG) of styrene onto ECTFE membranes. As the grafting amount of sulfonated styrene increased, the hydrophilicity of the membrane increased, and the absorption and permeability of water and methanol also increased.

The above research indicates the importance of solvents to the grafting rate during grafting. The cost and performance of sulfonated styrene-grafted ECTFE membranes are lower than those of Nafion. Therefore, ECTFE membranes grafted with sulfonated styrene have the potential to replace Nafion in direct methanol fuel cells.

### 4.5. ECTFE Membrane for Oil–Water Emulsion Separation

With the development of industrial processes such as petrochemicals, iron and steel, coking, and mechanical processing, the generation of oily wastewater has also increased substantially, and it has a long-term harmful effect on the ecological environment. Therefore, the separation of oil–water mixtures has received increasing attention [76,77,78,79,80]. The selection of membrane materials with certain surface wettability is the key to the application of membrane separation technology in oil–water separation. Membrane materials are categorized into hydrophobic and lipophilic surfaces and hydrophilic and lipophilic surfaces. Among them, the first two categories are the most widely studied for “degreasing” and “dehydration” processes. Pan [37] used the TIPS method to prepare superhydrophobic and super lipophilic ECTFE membranes by adding hydrophobic inorganic particles to the polymer solution and using the membranes for oil–water emulsion separation.

The membrane showed fine permeability and selectivity for kinds of surfactant-free and surfactant-stabilized water-in-oil emulsions. More importantly, the membrane exhibited excellent antifouling performance for long running in various pH conditions.

The compositions of polymer solutions, average pore size, and porosity of the membranes are shown in Table 9.

With increasing SiO_2_ content, the structure of the membrane surface changed gradually from hierarchical micro-/nanoporous to compact pores, and the average pore size and proportion of micropores in each membrane both gradually decreased.

To achieve superwettability of the membrane, a prerequisite is to obtain a rough surface, and then the design of a hierarchical micro-/nanostructure of the membrane surface will further enhance its wetting behavior. With the increase in SiO_2_ concentration, the size of protrusions became bigger and bigger, even forming more and more microspheres, which blocked the membrane pores and resulted in a decrease in surface porosity and pore size. Furthermore, it can be seen from the enlarged photograph of the membrane surface that the surface of the formed microspheres was not smooth but scattered all over with smaller-sized protrusions, like the protrusions on the surface of a lotus leaf. The cross sections of the prepared membranes changed from a bicontinuous to a stacking spherulitic structure with increasing SiO_2_ concentration. This is because the added SiO_2_ particles served as crystal nuclei and played a role in speeding the process of solid–liquid phase separation, causing the formation of spherulites instead of a bicontinuous pore structure with incomplete liquid–liquid phase separation during the forming process of the membrane. So, the membrane’s mean pore size and porosity decreased gradually, while the number and compactness of spherulites increased.

The above research indicates that the membrane has good permeability and selectivity to various water-in-oil emulsions without surfactants and stabilized surfactants. More importantly, the membrane also exhibits excellent antifouling performance under various hydrogen potential (pH) conditions for long-term operation. Therefore, the ECTFE membrane has great application prospects in the field of oil–water separation.

## 5. Conclusions and Perspectives

Overall, ECTFE membranes are promising materials with excellent chemical, thermal, and mechanical properties due to their unique structure, excellent corrosion resistance to many common chemicals, and stability under various harsh environmental conditions. Therefore, these membranes can be used in a variety of processes, such as in membrane condensers, organic-solvent filtration, PEMFCs, and oil/water emulsion separation of oil and water emulsions. Although some challenges remain, this old material is attracting more attention as more innovative ideas emerge. In research on the preparation of ECTFE membranes, it has become increasingly important for researchers to choose green solvents as diluents instead of traditional toxic solvents. Usually, surface grafting and surface oxidation techniques are used to treat the surface of ECTFE membranes to better meet application requirements.

Due to the difficulty of dissolving ECTFE material, there is limited research on ECTFE membranes. At the same time, diluent safety, production costs, and carbon emission issues put pressure on the environment. Therefore, finding a suitable diluent is crucial for the preparation of ECTFE membranes. Nevertheless, electrostatic spinning technology has reached a level of maturity. This approach, known for its simplicity, versatility, and continuous production of nanofibers, utilizes classic surface repulsion and viscous fluid as raw materials. it can generate fibers with diameters ranging from tens to hundreds of nanometers, exhibiting high porosity, a substantial specific surface area, diversified compositions, and uniform diameter distribution. Notably, electrospinning technology has already proven successful in producing membranes from insoluble polymers like polytetrafluoroethylene (PTFE). Therefore, the use of the electrostatic spinning method to prepare high-performance ECTFE membranes is an important future development direction of ECTFE.

## Figures and Tables

**Table 1:** ECTFE membrane fabrication by TIPS method and their applications.

Author	Polymer Type	Diluent Type	Diluent Boiling Point (°C)	Membrane Type	Membrane Process	Year
Ramaswamy [11] et al.	HALAR^®^901	Dibutyl phthalate (DBP)	337	Flat	-	2002
Roh [12] et al.	HALAR^®^901	Dibutyl phthalate (DBP)	337	Flat	-	2010
Simone [15] et al.	HALAR^®^901	1-methyl-2-pyrrolidinone (NMP)	202	Flat	Pervaporation	2012
Drioli [33] et al.	HALAR^®^901	Glyceryl triacetate (GTA)	258	Flat	Membrane condenser	2014
Pan [34] et al.	HALAR^®^902	Diethyl adipate (DEHA)/diethyl phthalate (DEP)	247/294	Flat	Membrane distillation	2015
Abdel-Hady [35] et al.	Commercial ECTFE membrane	Grafting of vinyl pyrrolidone (NVP)/styrene	-	Flat	Fuel cell	2015
Zhou [14] et al.	HALAR^®^902	Dibenzylidene sorbitol (DBS)/triphenyl phosphate (TPP)	549/412	Hollow fiber	-	2012
Matsuyama [36] et al.	HALAR^®^901	Diethyl phthalate (DEP) and glyceryl triacetate (GTA)	294/258	Hollow fiber	-	2016
Ursino [37] et al.	LMPECTFE	Diethyl phthalate (DEP)	294	Flat	Organic-solvent filtration separation	2016
Pan [38] et al.	HALAR^®^902	Diethyl adipate (DEHA)/diethyl phthalate (DEP)	247/294	Flat	Oil/water separation	2017
Abdel-Hamed [39] et al.	ECTFE-g-PSSA	-	-	Flat	Proton exchange membrane fuel cells (PEMFC)	2018
Xu [5] et al.	HALAR^®^902	Acetyl tributyl citrate (ATBC)	327	Flat	Membrane distillation	2019
Liu [40] et al.	HALAR^®^902	Trioctyl trimellitate (TOTM)	414	Flat	Membrane distillation	2020

**Table 2:** Interaction parameters between ECTFE and different solvents [14]. Copyright permission has been obtained from the Publisher Editorial Office.

Substance	Molar Volume (cm^3^·mol^−1^)	Solubility Parameter (MPa^1/2^)	Flory–Huggins Interaction Parameter/χ
Ethylene-chlorotrifluoroethylene (ECTFE)	-	17	-
Dimethyl phthalate (DMP)	163.46	22	1.80
Diethyl phthalate (DEP)	198.78	21	1.22
Dibutyl phthalate (DBP)	267.12	20	0.85
P-bromobenzenesulfonyl (BS)	387.03	18	0.37
Dibenzylidene sorbitol (DBS)	365.59	18	0.34

**Table 3:** Solubility parameters between ECTFE and different nonsolvents [41]. Copyright permission has been obtained from the Publisher Editorial Office.

Substance	Molar Volume (cm^3^·mol^−1^)	Solubility Parameter (MPa^1/2^)	Flory–Huggins Interaction Parameter/χ
Dibenzylidene sorbitol (DBS)	337	19	0.64
Hexamethylenediaminocarbamat (HDC)	299	18	0.37
Amine oxide (OA)	316	19	0.84
Triphenyl phosphate (TPP)	262	19	0.65
Triethylene glycol (TEG)	173	20	0.97

**Table 4:** The effect of various diluting solvents on the degree of grafting (DG) of styrene onto ECTFE films [39].

Solvent	DG (%)
Chloroform	8
Tetrahydrofuran	8
1,2-dichloroethane	9
Toluene	11
Methanol + methylene chloride 1:1	21.3

**Table 5:** Average pore size and porosity of ECTFE membranes prepared at different polymer concentrations (quenching temperature: 20 °C) [5].

ECTFE Concentrations	Average Pore Size (nm)	Porosity (%)
20	472 ± 20.3	65.2 ± 4.5
25	327 ± 18.2	58.5 ± 3.3
30	316 ± 19.3	50.1 ± 2.1
40	115 ± 10.9	31.9 ± 2.5

**Table 6:** Average pore size and porosity of ECTFE membranes prepared at different quenching temperature (polymer concentrations: 30 Wt.%) [5].

Quenching Temperature	Average Pore Size (nm)	Porosity (%)
20	316 ± 19.3	50.1 ± 2.1
40	239 ± 15.6	55.6 ± 2.3
60	223 ± 11.5	52.8 ± 3.3
80	153 ± 10.8	55.2 ± 3.6

**Table 7:** VMD performance comparison of ECTFE membrane in this study and the literature.

Reference	Diluent	Application	Permeation Flux (L/(m^2^.h))	Salt Rejection (%)	Feed Solution	Feed Inlet Temperature (°C)
[34]	Diethyl adipate (DEHA)/diethyl phthalate (DEP)	Vacuum membrane distillation (VMD)	16.7	99.9	3.5% Wt NaCl solution	80
[5]	Acetyl tributyl citrate (ATBC)	Vacuum membrane distillation (VMD)	22.3	99.9	3.5% Wt NaCl solution	80

**Table 8:** Properties of the LMP ECTFE membranes [37].

Membrane	Average Pore Size (μm)	Porosity (%)
L2	0.03	69.5
M2	0.01	56
N2	0.01	42.3

**Table 9:** The composition, average pore size, and porosity of ECTFE hybrid porous membranes.

Sample	ECTFE (Wt.%)	DEHA (Wt.%)	DEP (Wt.%)	SiO_2_	Average Pore Size (nm)	Porosity (%)
M-S0	20	18	62	0	543	74
M-S2	20	18	62	2	375	72
M-S4	20	18	62	4	242	71
M-S6	20	18	62	6	191	71
M-S8	20	18	62	8	123	67

## Data Availability

No new data were created or analyzed in this study. Data sharing is not applicable to this article.
